# Airborne Particulate Matter as an Emerging Driver of Gastric Carcinogenesis: Molecular Pathways Linking Inflammation and Cancer

**DOI:** 10.3390/ijms27125203

**Published:** 2026-06-09

**Authors:** Yesennia Sánchez-Pérez, Yanueh Bautista-Ocampo, Edith Moreno-Bautista, Rocío Morales-Bárcenas, Raúl Quintana-Belmares, Marytere Herrera-Martínez, Jossimar Coronel-Hernández, Dennis Cerrato-Izaguirre, Claudia M. García-Cuellar, Ericka Marel Quezada-Maldonado

**Affiliations:** 1 Subdirección de Investigación Básica, Instituto Nacional de Cancerología, San Fernando No. 22, Tlalpan, Ciudad de Mexico CP 14080, Mexico; s_yesennia@yahoo.com.mx (Y.S.-P.); heunay@hotmail.com (Y.B.-O.); edithmoreno2201@gmail.com (E.M.-B.); mobarobiol@yahoo.com.mx (R.M.-B.); qbro76@gmail.com (R.Q.-B.); jossithunders@gmail.com (J.C.-H.); dennis_cerrato@yahoo.com (D.C.-I.); 2 Unidad Funcional de Gastroenterología, Instituto Nacional de Cancerología, San Fernando No. 22, Tlalpan, Ciudad de Mexico CP 14080, Mexico; mherrera_84@hotmail.com; 3 Dirección de Investigación, Instituto Nacional de Cancerología, San Fernando No. 22, Tlalpan, Ciudad de Mexico CP 14080, Mexico

**Keywords:** particulate matter, gastric cancer, carcinogenesis, inflammation

## Abstract

Gastric cancer (GC) remains a leading cause of cancer-related mortality worldwide, with chronic inflammation playing a central role in its pathogenesis. While established risk factors such as Helicobacter pylori (Hp), diet, and lifestyle are well recognized, growing epidemiological evidence links airborne particulate matter (PM) exposure with increased GC incidence and mortality. However, the biological mechanisms underlying this association remain poorly understood. This review integrates epidemiological evidence associating elevated PM exposure with GC risk and summarizes current mechanistic knowledge regarding PM gastric translocation and retention. The influence of PM size, chemical composition, and surface reactivity on biological activity is also discussed, highlighting the stomach as a plausible yet understudied target organ. Additionally, we compiled evidence from studies published between 2010 and 2026 demonstrating the ability of PM to induce inflammatory responses through activation of NF-κB, MAPK, JAK/STAT, and COX-2 signaling pathways across diverse biological systems. Although PM-induced inflammation has been extensively characterized in respiratory and other tissues, its contribution to gastric carcinogenesis remains largely unexplored. We propose that PM exposure may exacerbate Hp-driven inflammation, promoting a persistent pro-inflammatory microenvironment conducive to tumor initiation and progression. Collectively, these findings position PM as a biologically plausible and potentially modifiable risk factor for GC.

## 1. Introduction

Gastric cancer (GC) represents a critical challenge to global health, establishing itself as one of the most prevalent neoplasms with high mortality rates, primarily due to late diagnosis at advanced stages. Its etiology is multifactorial, arising from the complex interplay between host genetic susceptibility and persistent exposure to multiple risk factors [1]. Emerging epidemiological evidence indicates that particulate matter (PM) is a critical, yet underrecognized, environmental risk factor [2]. Despite the increasing global incidence of this neoplasm, the link between air pollution and gastric carcinogenesis remains insufficiently explored. This review integrates epidemiological findings in GC with the experimental characterization of three key inflammatory pathways that, given the limited direct evidence in gastric tissue, have been documented primarily in other organ models, but are known to have a critical and well-established impact on gastric carcinogenesis. By detailing how PM induces a persistent inflammatory microenvironment through these molecular effects, this review seeks to elucidate one of the mechanisms that could drive malignant transformation in the stomach, highlighting an urgent yet underreported area of research.

## 2. Global Overview of Gastric Cancer

Gastric cancer (GC) is the fifth most diagnosed cancer and leading cause of cancer death worldwide, with over 968,000 cases and 660,000 deaths in 2022 [3]. Incidence varies by region—concentrated in Eastern Asia, Eastern Europe, and Latin America—and depends on modifiable factors like *Helicobacter pylori* (Hp) and diet [4]. Five-year survival is below 30% in most countries due to late diagnosis, except in Korea (69%) and Japan (60%), where early detection is standard [4,5]. GC etiology involves intrinsic and environmental interactions. GC originates primarily in the gastric epithelium. In 95% of cases, it is presented as adenocarcinoma, classified into intestinal and diffuse subtypes; the latter being more aggressive [6].Clinically, tumors are divided by location: Cardia GC originates in the proximal region adjacent to the esophagus, increasing in Western nations, links to obesity and reflux; whereas non-cardia GC (from the distal segments of the stomach, including the fundus, body, antrum and pylorus), prevalent in developing countries, is strongly associated with Hp and high-salt diets [7,8,9].

## 3. Risk Factor of GC

### 3.1. Non-Modifiable GC Risk Factors

In the etiology of GC, non-modifiable risk factors, defined as biological or intrinsic variables beyond an individual’s preventive control, play a determining role. Incidence shows a marked gender disparity, being twice as frequent in men [3]. While historically associated with older adults, an upward trend is now observed in adults under 50 [10,11]. Genetic predisposition occurs in 10% of cases, though only 1–3% are strictly hereditary [12]. The *CDH1* germline mutation is the most relevant alteration, significantly increasing the risk of hereditary diffuse gastric cancer. Other syndromes increasing susceptibility include Lynch, Peutz-Jeghers, Li-Fraumeni, hereditary breast and ovarian cancer, familial adenomatous polyposis, and gastric adenocarcinoma with proximal polyposis. These entities underscore the genetic heterogeneity of inherited GC susceptibility [13,14].

### 3.2. Traditional GC Risk Factors

Lifestyle and environmental factors significantly drive GC development. Hp is the primary oncogenic agent, causing 90% of gastric adenocarcinomas [9,15]. Persistent infection induces chronic gastric mucosal inflammation, leading to pathological processes, such as Correa cascade, a histopathological sequence that progresses from chronic gastritis to atrophy, intestinal metaplasia, dysplasia, and adenocarcinoma [16]. Furthermore, the Epstein-Barr virus (EBV) accounts for 8% of cases, exhibiting a unique molecular profile marked by extreme DNA hypermethylation of CpG islands of tumor suppressor genes like *CDH1*, *CDKN2A* and *RUNX3* [17]. Complementing the infectious etiology, dietary risks include high salt intake, processed foods with N-nitroso compounds, and low fruit/vegetable consumption [18,19]. Lifestyle habits also catalyze carcinogenesis: smoking introduces systemic carcinogens, alcohol disrupts the mucosal barrier, and obesity promotes chronic low-grade inflammation and adipokine dysregulation, which alters the gastric microenvironment [20,21,22]. Occupational and environmental exposures, often overlooked, are critical. Exposure to mining dust, ionizing radiation, and heavy metals like arsenic, cadmium, lead and chromium, significantly increases GC risk [23,24,25,26,27]. Recently, ambient air pollution, specifically particulate matter (PM), has emerged as a key concern due to its toxic organic compounds and metals [28,29,30]. Consequently, understanding the role of PM in gastric oncogenesis is paramount. Despite the rising global incidence of gastric cancer, the association between air pollution and gastric carcinogenesis remains incompletely characterized.

## 4. Air Pollution, Another Risk Factor for GC

### 4.1. Types of Particulate Matter, Composition and Carcinogenicity (PM_10_, PM_2.5_, PM_1-0.1_)

The Global Burden of Disease Study identifies air pollution as the second leading cause of death worldwide, causing approximately 4.9 million premature deaths annually with PM being its most harmful component [31,32]. World Health Organization (WHO) data indicate that over 90% of the global population resides in areas exceeding air quality guidelines [33]. PM is a complex heterogeneous mixture of elemental carbon, inorganic compounds (e.g., salts, metals), organic components (e.g., dioxins and polycyclic aromatic hydrocarbons (PAHs)), and biological elements (e.g., endotoxins and microorganisms) [34,35]. According to its size fraction, PM is classified into PM_10_ (aerodynamic diameter ≤ 10 µm), PM_2.5_ (aerodynamic diameter ≤ 2.5 µm), and ultrafine particles (UFP, aerodynamic diameter ≤ 0.1 µm), these comprise the inhalable fraction capable of reaching the deep respiratory tract [36,37]. While the International Agency for Research on Cancer (IARC) classifies PM as a Group 1 carcinogen, based on its link to lung cancer [38,39]. Recent epidemiological evidence suggests its effects extend systemically. Chronic exposure is now associated with increased risks of distal tumors, including bladder, breast, colorectal, and gastric cancer [40], indicating that PM components play a critical role in extrapulmonary carcinogenesis.

Due to its physicochemical heterogeneity, PM interacts with multiple biological systems, primarily through oxidative stress, inflammation, and genotoxicity. At the cellular level, PM-derived transition metals and PAH metabolites generate reactive oxygen species (ROS), damaging lipids, proteins, and DNA. These events activate stress-sensitive signaling pathways that promote proinflammatory cytokines, chemokines, and cell survival mediators [41,42,43]. Furthermore, PM alters cell death mechanisms, DNA repair, and the epithelial-mesenchymal transition [44,45,46]. As a multifaceted toxic agent, PM disrupts cellular homeostasis and promotes persistent pathological states linked to carcinogenesis. The following section synthesizes the epidemiological evidence linking PM exposure to GC, providing crucial context for understanding the underlying mechanisms.

### 4.2. Epidemiological Evidence of the Effect of PM on GC

One of the first epidemiological studies of the potential association between PM exposure and GC found that mortality from this neoplasm was up to twice as high in areas with elevated PM concentrations than in those with lower PM levels. This initial finding was crucial in establishing that air pollution is a relevant environmental factor in the etiology of this disease [47]. Despite these initial findings, evidence was limited for decades. More recently, a Taiwanese study analyzing deaths in Taiwan over a four-year period confirmed that PM exposure contributes to the risk of gastric cancer incidence and mortality. The risk of death from gastric cancer increased by 26% (Odds ratio [OR]: 1.26; 95% CI: 1.04–1.53) among individuals residing in areas with high traffic-related air pollution exposure, estimated using gas station density as a proxy for exposure to benzene and PAHs, important constituents of PM. This association suggests a potential role of traffic-derived airborne pollutants in gastric carcinogenesis. This suggests that pathogenesis is likely related to the systemic absorption of these pollutants, either through inhalation or ingestion [48].

Other studies have suggested that a three-year period of PM_2.5_ exposure is significantly associated with mortality from GC with a Relative Risk (RR) of 1.0003 (95% CI; 1.0001, 1.002), highlighting that the impact of pollution is not immediate but manifests itself with a time delay [49]. Additionally, each 1 μg/m^3^ increase in PM_2.5_ exposure is significantly associated with a RR 1.028 (95% CI: 1.011, 1.046) for GC mortality over five years [50]. In a seventeen-year population follow-up study, an association was observed for each 10 µg/m^3^ increase in PM_2.5_ with an 87% higher risk of CG mortality (Hazard ratio (HR): 1.87, 95% CI: 1.20–2.92) in the overall cohort and with a twofold increased risk in never-smokers (HR: 2.01; 95% CI: 1.01–3.98). However, these associations lost statistical significance after adjustment for multiple comparisons [51].

The most recent evidence suggests that even acute exposure to PM can increase the risk of CG mortality. Yu et al. reported that for every 10 μg/m^3^ increase in average PM_2.5_ exposure over three days, the OR for gastric mortality was 1.05 (95% CI: 1.02–1.08) [52]. Complementarily, another study found that a similar increase in PM_2.5_ was associated with a RR increase of 1.011 (95% CI: 1.000–1.022) for GC, and importantly, PM_10_ also showed a significant effect (RR: 1.009; 95% CI: 1.001–1.017). Furthermore, this study noted a greater impact on men and older adults, indicating increased vulnerability in these groups [2]. In the context of individual components presents in PM, an association analysis of long-term exposure to PM_2.5_ (ten years) showed that for every 0.46 μg/m^3^ increase in black carbon concentration, mortality from CG showed a significant increase of 37% (RR: 1.37 95% CI: 1.26, 1.49) and 33% (RR: 1.33 95% CI: 1.23, 1.45) for each 4.56 μg/m^3^ increase of organic carbon, with the effect being mainly observed in men [53].

About the incidence of GC, the European multicenter ESCAPE study found that prolonged exposure to PM_2.5_ is associated with an increased risk of developing gastric cancer. Specifically, for every 5 µg/m^3^ increase in PM_2.5_ concentration, the risk increased by 38% (HR: 1.38; 95% CI: 0.99–1.92). Furthermore, the study identified sulfur present in these particles as the component most strongly associated with this increased risk (HR: 1.93; 95%, CI: 1.13–3.27) [54,55]. An average concentration of PM_2.5_ over two years was found to be significantly associated with an increased risk of gastric cancer. Specifically, for every 10 µg/m^3^ increase in baseline PM_2.5_ concentration, the risk of developing this cancer increased by 27% (HR: 1.27; 95% CI: 1.02–1.58) [56]. A positive correlation was also identified between PM_1_ exposure and GC incidence (RR: 1.023; 95% CI: 1.007–1.039), in a specific region of northwest China, characterized by a high prevalence of this neoplasm. Consistent with other studies, it has been suggested that men may be more susceptible to PM_1_ exposure [57]. Collectively, these studies reveal a consistent association between particulate matter exposure and both gastric cancer incidence and mortality across different populations and exposure windows.

## 5. Mechanisms of PM Translocation to Stomach: Systemic Circulation, Ingestion, and Mucociliary Clearance

Pollutant entry depends on physicochemical properties and exposure routes, primarily inhalation, ingestion, and dermal absorption. Bioaccumulation and distribution depend on size, solubility, and the affinity of lipidic and protein compounds. PM poses a systemic risk due to their ability to penetrate deep into the respiratory tract and translocate from alveoli into the bloodstream and extrapulmonary organs [58,59]. Particle size determines internal distribution: PM_10_ typically deposits in the tracheobronchial region, while PM_0.1_ can reach the alveolar region and enter systemic circulation via diffusion thus reaching distal organs [60,61].

Experimental models and human studies confirm this systemic spread [62,63]. Respiratory instillation of PM in rats results in accumulation in distal organs, including the brain, heart, liver, and kidneys and testicles [64]. Furthermore, mucociliary clearance acts as a critical pathway for gastrointestinal exposure. Inhaled particles captured by mucus and macrophages in the airways are transported to the pharynx by ciliary action and subsequently swallowed. Consequently, this mechanism ensures that a significant fraction of inhaled PM reaches the gastrointestinal tract, expanding the scope of toxicological impact beyond the respiratory system [65].

PM composition also determines its distribution. The translocation of PM-associated metals into systemic circulation depends on their solubility in aqueous or acidic media. In animal models, water-soluble metals like vanadium and nickel translocate rapidly to the plasma, heart, and liver, whereas low-solubility elements like silicon remain confined to the lungs [66]. The gastrointestinal tract is a critical absorption route for these components. Comparative analyses in simulated biological fluids show that gastric juice solubilizes metals, such as lead, at a significantly higher rate than pulmonary or interstitial fluids, especially for particles < 20 µm [67]. Furthermore, the acidic pH of the stomach enhances the bioaccessibility of heavy metals in PM_10_ increasing their bioavailability and enabling direct interaction with the gastric epithelium [68,69].

Oral ingestion is a critical exposure route, as PM deposits significantly in food and water. Metals (Cd, Cu, Cr, Pb, Zn, As, Ni) in PM_2.5_ are highly bioaccessible under gastrointestinal pH [70], with oral bioaccessibility reaching up to 100%, compared to an average of 45% in the respiratory tract [71,72]. PAHs also exhibit measurable bioaccessibility in simulated gastric conditions, with 2–31% released and a 10% bioavailable fraction. While this proportion is lower than that of metals (40–75%), it remains biologically significant due to the potent mutagenic and carcinogenic properties of PAHs. Under chronic exposure scenarios, even limited release can lead to meaningful cumulative toxicological effects [73,74,75].

PAH bioaccessibility is significantly influenced by the gastrointestinal microenvironment. Dietary proteins and lipids enhance the solubilization of hydrophobic compounds, facilitating their desorption from particles and increasing epithelial absorption. These findings underscore the gastrointestinal tract as a critical, underrecognized route for particle-bound toxicants. Whether via direct ingestion or mucociliary clearance, the gastric epithelium acts as a primary interface for toxicant interaction. Consequently, evaluating air pollution health risks must incorporate gastrointestinal exposure as a pivotal mediator of carcinogenesis [75,76].

## 6. Overview of Inflammatory Pathways as a Central Axis in GC

Inflammation is central to carcinogenesis, driving transformation, survival, and metastasis. It arises from external factors (infections, smoking, pollutants) or intrinsic genetic alterations that recruit inflammatory cells [77]. Chronic inflammation causes sustained tissue damage, activating repair mechanisms and epithelial proliferation [78]. In the stomach, the inflammatory process triggers the Correa cascade: a sequence from chronic gastritis to atrophy, intestinal metaplasia, dysplasia, and adenocarcinoma. While metaplasia is a reversible adaptive change, persistent stimuli lead to dysplasia, atypical cellular growth and lost architecture marking the direct precursor to invasive carcinoma [79,80]. This inflammatory state is primarily maintained by the NF-κB, MAPK, and JAK/STAT signaling pathways, which are detailed below.

### 6.1. NF-κB as a Master Regulator of Gastric Inflammation

Nuclear factor kappa B (NF-κB) is a family of transcription factors (*RelA* (p65), *RelB*, *c-Rel* (REI), *NFκB1* (p50), and *NFκB2* (p52)), that remains inactive in the cytoplasm under basal conditions through its interaction with IκB inhibitory proteins. In response to inflammatory stimuli and exogenous agents or pathogens IκB proteins are degraded, allowing NF-κB to translocate to the nucleus and activate the transcription of multiple genes including cytokines, anti-apoptotic proteins, and VEGF [81,82]. Activation of NF-κB contributes to tissue damage and increases the risk of tumorigenesis [83]. The NF-κB signaling pathway plays a key role in GC initiation and progression by regulating inflammation, cell survival, proliferation, and immune evasion. Its persistent activation, commonly associated with Hp and EBV infections, promotes tumor progression, aggressive phenotypes, and resistance to therapy [84,85,86]. Moreover, increased NF-κB expression has been associated with poor overall survival in GC patients [84,87].

### 6.2. MAPK Pathway: Is a Key Node in the Integration of Inflammatory Signals

The mitogen-activated protein kinase (MAPK) pathway is a key signaling cascade activated in response to external stimuli and environmental stress, regulating the release of inflammatory mediators, cytokines, and growth factors [88]. This pathway involves a sequential phosphorylation cascade (MAPKKK (Raf)–MAPKK (MEK)–MAPK), leading to the activation of major kinases such as Extracellular Signal-Regulated Kinases (ERK), c-Jun N-terminal Kinases (JNK1/2/3), and p38 mitogen-activated protein kinase (p38α/β/γ/δ). These kinases regulate transcription factors including NF-κB and AP-1, thereby controlling gene expression related to inflammation, metabolism, proliferation, and stress responses [89,90]. MAPK signaling pathways play a central role in GC development and progression. Their hyperactivation, observed in approximately 30% of gastric tumors, promotes tumor progression through ERK1/2 activation and stress-related pathways such as JNK and p38, which contribute to inflammation and genomic instability [88,91]. Moreover, MAPK activation has been associated with shorter overall survival, metastasis, and therapeutic resistance, highlighting its key role in gastric carcinogenesis [92,93].

### 6.3. JAK/STATs as the Central Axis of Inflammation and Oncogenesis

The Janus Kinase/Signal Transducer and Activator of Transcription (JAK/STAT) signaling pathway is a key mechanism that translates inflammatory signals into transcriptional responses. Upon cytokine binding, JAK kinases (JAK1, JAK2, JAK3, and TYK2) are recruited and phosphorylate STAT proteins (STAT1, STAT2, STAT3, STAT4, STAT5a, STAT5b, and STAT6), which then translocate to the nucleus to regulate genes involved in cell survival, proliferation, angiogenesis, and immune responses. Dysregulation of this pathway during chronic inflammation promotes a pro-tumorigenic microenvironment by inhibiting apoptosis and increasing genomic instability [94,95]. The JAK/STAT signaling pathway, particularly STAT3, plays a key role in gastric cancer development and progression by mediating responses to inflammatory stimuli [96]. Its persistent activation, commonly associated with Hp and EBV infections, promotes tumor growth, metastasis, and maintenance of cancer stem-like cells. Moreover, elevated JAK/STAT activity has been linked to poor prognosis, reduced survival, and chemotherapy resistance, highlighting its relevance as a potential therapeutic target [97,98,99].

### 6.4. COX-2/PGE2 Pathway: A Crosstalk Between Multiple Inflammation Pathways and Tumor Promotion

Cyclooxygenase-2 (COX-2) is an inducible enzyme that acts as a central convergence point for inflammatory and environmental signals. Its expression is regulated by multiple pathways, including NF-κB, MAPK, and JAK/STAT, which cooperate to induce and sustain *PTGS2* (COX-2) transcription [100]. While NF-κB initiates COX-2 expression, MAPK signaling stabilizes its mRNA, and JAK/STAT further enhances its transcription, creating a positive feedback loop that maintains a pro-inflammatory and pro-tumorigenic microenvironment [101,102]. Functionally, COX-2 catalyzes the production of prostaglandins, particularly PGE_2_, which promotes tumorigenesis by indirectly activation of downstream pathways such as EGFR, MAPK, PI3K/AKT, and Wnt/β-catenin. These signaling networks collectively enhance cell proliferation, survival, angiogenesis, immune evasion, invasion, and metastasis. Consequently, in gastric cancer, COX-2 overexpression has been consistently associated with tumor progression and has been proposed as a prognostic marker and a potential therapeutic target [103,104,105,106,107].

## 7. Evidence of the Role of PM as a Modulator of Inflammatory Signaling Pathways

### 7.1. PM and NF-κB Activation

Evidence shows that PM induces aberrant NF-κB activation. In lung epithelial cells (16HBE) PM_2.5_ exposure reduces viability, and triggers morphology alterations, and apoptosis through the TLR4/NF-κB pathway, primarily driven by associated lipopolysaccharides (LPS) [108]. Consistently, this same signaling mechanism promoted NF-κB activation in microglial cells exposed to PM [109]. PM_2.5_ triggers a robust inflammatory response via ROS production and the nuclear translocation of NF-κB p65. This activates the NLRP3 inflammasome, upregulating proinflammatory cytokines (IL-6, TNF-α, IL-1β, IL-18) and key mediators such as iNOS, COX-2, caspase-1, and GSDMD [109,110,111,112]. Additionally, PM disrupts the PI3K/Akt pathway, a process potentially modulated by the TREM2 receptor, which regulates survival and inflammation in response to environmental pollutants [110].

Exposure to PM_2.5_ increases pro-inflammatory mediators and activates Nod2, NF-κB p65 and NLRP3 in alveolar macrophage (MH-S); notably these effects are attenuated by irisin, a polypeptide derived from the hydrolysis of the fibronectin type III domain-containing protein 5 (FNDC5) [113]. In murine models, respiratory administration of PM induces myocardial apoptosis, characterized by caspase-3, Bax, and Bcl-2 dysregulation and promotes lung inflammation via the IL-6/AKT/STAT3/NF-κB axis, upregulating intercellular adhesion molecule-1 (ICAM-1) [114,115]. Furthermore, cutaneous exposure to PM_2.5_ triggers TLR5 and NOX4 activation, leading to NF-κB phosphorylation, nuclear translocation, and increased IL-6 expression. These molecular changes result in heightened oxidative stress and histopathological evidence of skin inflammation [116]. Antioxidants use prior to PM_2.5_ exposure prevents NF-κB activation, suggesting the role of PM-associated metals in ROS generation and transcriptional upregulation [109,115]. Additionally, non-coding RNAs non-coding RNAs modulate this inflammatory response; specifically, PM exposure downregulates miR-140-5p and miR-149-5p while upregulating miR-146a in various lung cell lines [108,117,118].

### 7.2. PM and MAPK Activation

PM consistently triggers respiratory alterations via MAPK signaling pathways (ERK1/2, p38, and JNK). In in human bronchial epithelial cells (HBECs) and lung tissues, PM_2.5_ induced ROS production phosphorylates these kinases, leading to inflammatory infiltration and upregulated expression of proinflammatory cytokines (IL-1β, IL-6, IL-8) and mucus-related genes (MUC5AC) [119,120,121]. In mice, airway barrier disruption is regulated by the phosphatase Shp2 through ERK1/2-MAPK activation; notably, antioxidants like N-acetylcysteine effectively inhibit this response [122]. Consistently, chronic exposure to PM_10_ in rat maintains sustained ERK1/2 activation, associated with pulmonary arteriolar hyperreactivity and vascular remodeling and muscarinic receptor (CHRM3) overexpression [123,124,125]. Beyond the respiratory system, PM-induced ROS in corneal cells promote p38 phosphorylation, NF-κB translocation, mitochondrial damage, and DNA double-strand break [126].

Furthermore, PM positively regulates endothelin receptors in rat basilar arteries [127] and activates p38/NF-κB in human keratinocytes (HaCaT), upregulating transient receptor potential vanilloid 1 gene (TRPV1) and activator protein-1 (AP-1). These events decrease cell proliferation and increase inflammatory responses, contributing to conditions like premature skin aging [128,129]. In macrophages (RAW 264.7) PM in combination with BaP induces TNF-α secretion, activating the MEK1/2 cascade and ERK1/2 (p42/p44) phosphorylation, ultimately promoting apoptosis [130]. Similarly, in cardiovascular models (endothelial cells (EA.hy 926 and HUVECs), cardiomyocytes (H9c2), and arteries) PM_2.5_ increase intracellular ROS, promoting phosphorylation of ERK1/2, p38 and JNK. This MAPK activation is associated with an exacerbated pro-inflammatory response, decreased cell viability and the induction of ICAM-1 and VCAM-1, alongside thrombotic events [131,132,133,134,135,136]. Together these findings establish the MAPK pathway as a critical axis linking PM exposure to oxidative stress, cardiovascular inflammation, and endothelial dysfunction.

PM induces selective epigenetic reprogramming of the MAPK pathway. Short-term exposure leads to promoter methylation changes across multiple pathway genes in peripheral blood leukocytes [137]. Furthermore, PM promotes airway inflammation in bronchial epithelial cells by increasing histone deacetylase 9 (HDAC9) levels which deacetylates histone 4 at K12 (H4K12) at the dual specificity phosphatase 9 (DUSP9) promoter. This represses DUSP9 expression, resulting in sustained MAPK signaling activation both in vitro and in vivo [138].

In the case of non-coding RNA, PM_2.5_ exposure drives microglia toward a pro-inflammatory phenotype by secreting extracellular vesicles (EVs) with miR-34a-5p. This miRNA targets DUSP10, activating the p-p38 MAPK pathway, which increases aberrant tau phosphorylation and pathological Aβ protein production ultimately inducing neuro-apoptosis in hippocampal and cortical regions [139]. In lung cells (16HBE), PM-induced malignant transformation is linked to the downregulation of lncRNA SPRY4-IT1. Low levels of this lncRNA promote DUSP6 ubiquitination, leading to sustained ERK1/2 phosphorylation and activity. This mechanism accelerates cell cycle progression and proliferation, facilitating the carcinogenic process [140].

### 7.3. PM and JAK/STAT Activation

PM significantly impacts the JAK/STAT pathway. In lung cell lines (A549), PM_10_ induced phosphorylation of STAT3 at Tyr705 residue, mediated by the non-canonical pathway of specific Src and PKCzeta kinases, promoting apoptosis evasion [141]. While PM_2.5_ activates the JAK2/STAT3 axis through ROS production, upregulating IL-6, IL-8, and COX-2, which correlates with altered cell cycle progression and increased apoptosis [142,143,144]. These findings are consistent with in vivo models, where PM intratracheal instillation triggers analogous activation of JAK2/STAT3 or STAT5 in lung tissue. In addition, PM_2.5_ induces methylation of the TIPE2 promoter, silencing this critical negative regulator of inflammation [142,145]. PM also increased phosphorylation of JAK1, JAK2, and STAT6, indicating that activation of the JAK/STAT6 pathway plays a central role in regulating eosinophil recruitment and amplifying the inflammatory response in mice lungs, primarily through increased IL-13 production. Notably, sustained activation of this signaling pathway under continuous PM exposure conditions (27 days) is associated with the most severe inflammatory effects, in contrast to those observed after short-term exposures [146].

Cardiomyocytes exposed to PM_2.5_ exhibit significant increase in JAK/STAT3 phosphorylation, increasing IL-6 and TNF-α, as well as the apoptotic index. These damage-mediating effects are notably prevented by 1,25-vitamin D_3_ [147]. In human vascular endothelial cells (HUVECs), PM_2.5_ increases IL-6 synthesis, which activate JAK1 kinase, through autocrine or paracrine signaling. This induces STAT3 phosphorylation (Tyr705) and its nuclear translocation, upregulating ICAM-1, VCAM-1, and E-selectin, thereby orchestrating endothelial activation and leukocyte recruitment [148]. Similarly, astrocytes exposed to PM show increased inducible nitric oxide synthase (iNOS) and IL-1β production, subsequently activating JAK2/STAT3 and p38/JNK/ERK MAPKs pathways [149].

The inorganic fraction of PM_10_ (especially Pb, followed by Zn and Cu) suppresses miR-26a, leading to LIN28B overexpression. This activates the IL-6/STAT3 axis in A549 cells, promoting epithelial-mesenchymal transition (EMT) and accelerating tumor migration and invasion. Additionally, PM_2.5_ exposure increases levels of LOC101927514, a lncRNA that interacts with the p-STAT3 protein in the nucleus to modulate multiple oncogenic pathways. In BEAS-2B cells, PM_2.5_ also elevate levels of the circular RNA circ406961; this molecule interacts with the ILF-2 protein to stabilize JAK/STAT3 pathway activation, exacerbating the release of pro-inflammatory mediators such as IL-6, IL-8, and TNF-α [150,151,152].

### 7.4. PM and COX-2 Activation as a Common Target of Inflammatory Pathways

Different studies clearly demonstrate that exposure to PM_2.5_ significantly increases COX-2 protein in macrophages (RAW264.7) and mouse aortic endothelial cells (MAECs). via TLR4 and NF-κB activation. This time-dependent response is accompanied by elevated IL-6, TNF-α, MCP-1, and iNOS, driving M1 polarization and positioning COX-2 as a key effector in systemic inflammation [153,154]. In human bronchial epithelial cells (16HBE) PM_2.5_ induces COX-2 through oxidative stress and activation of the JAK2/STAT3 pathway increasing IL-6 and IL-8 production. Notably, this inflammatory response is effectively abrogated by antioxidant treatment or pharmacological inhibition of JAK2 [144].

PM_2.5_ exposure increases COX-2 expression in primary cultured hippocampal neurons through ROS generation, leading to overproduction of PGE2 in hippocampal slices and excitatory neurotoxicity [155]. Similarly, in human gingival and synovial fibroblasts, PM triggers COX-2 and PGE2 release through the Akt, p38 MAPK, ERK1/2, and NADPH oxidase/ROS/NF-κB pathways; notably, resveratrol can inhibit these effects by blocking ROS activation [156,157]. In vivo models confirm that respiratory PM exposure elevates the COX-2/PGE2 axis in the bronchial epithelium via PI3K signaling. This activation directly contributes to epithelial barrier damage by decreasing the expression of filaggrin, a protein essential for membrane integrity [158].

## 8. Inflammation as a Common Mechanism of Gastric Cancer Risk Factors: Potential Contribution of Particulate Matter

In gastric cancer, extrinsic factors such as Hp infection, EBV, smoking, alcohol consumption, and diet induce epithelial damage, mucosal barrier disruption, and promote ROS generation. These events foster a chronic inflammatory microenvironment that promotes genomic instability and carcinogenesis [159,160,161,162,163]. At the molecular level, Hp infection, through virulence factors such as CagA, and EBV latent proteins, trigger sustained NF-κB pathway activation, upregulating pro-tumor mediators like COX-2. Simultaneously, tobacco nitrosamines activate the MAPK pathway, driving cell proliferation. Convergently, these stimuli activate the JAK/STAT axis (specifically STAT3), which integrates IL-6-mediated signals to promote apoptosis evasion, EMT, and tumor progression [164,165,166,167].

The interplay among risk factors is critical, as one can modulate the biological impact of others. For instance, tobacco and alcohol consumption increases the likelihood of persistent Hp infection and reduces the effectiveness of eradication therapies [168,169,170]. This synergy accelerates the progression of preneoplastic lesions by maintaining a state of persistent oxidative stress. Similarly, high-salt diets, ultra-processed foods, and grilled meats, rich in transition metals and PAHs, exacerbate bacterial inflammation and promote the formation of N-nitroso compounds, aggravating genotoxic damage. Together, this complex network overstimulates shared inflammatory pathways, transforming the gastric epithelium into a pro-oncogenic microenvironment [171,172].

**Figure 1:**
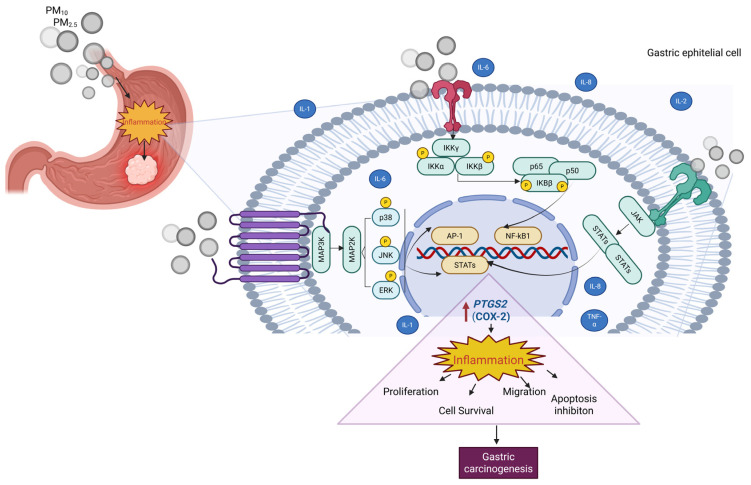
Schematic representation of the potential inflammatory pathways activated upon particulate matter (PM) contact with the gastric epithelium. In other organs, PM exposure has been shown to activate key signaling pathways such as NF-κB, MAPKs, and JAK/STAT. It is therefore plausible that similar mechanisms may occur in the gastric mucosa, leading to the upregulation of pro-inflammatory mediators, including COX-2, and the subsequent secretion of interleukins. Sustained activation of these inflammatory pathways may promote cellular processes such as proliferation, survival, migration, invasion, and evasion of apoptosis. Chronic inflammatory alterations of this nature could contribute to gastric carcinogenesis. Created in https://BioRender.com.

In the context of pre-existing vulnerability, exposure PM_10_ and PM_2.5_, could exacerbate the gastric inflammatory microenvironment. As established, PM activates the same key molecular pathways involved in gastric carcinogenesis induced by traditional risk factors, specifically NF-κB, MAPK, and JAK/STAT, which regulate chronic inflammation, proliferation, and apoptosis evasion (Figure 1). Consequently, continuous PM exposure in highly polluted areas could intensify pro-inflammatory signals, accelerating the accumulation of alterations that promote malignant progression. PM should therefore be integrated into GC risk assessment models and public health strategies as a relevant environmental factor influencing both the risk and biological evolution of the disease.

## 9. Discussion

Gastric cancer is a multifactorial disease in which, although factors such as Hp infection, smoking, and alcohol consumption have been widely studied [9,19,173], these do not fully explain the total heterogeneity in the increased incidence, progression, and aggressiveness of the disease, particularly in younger patients. In this context, identifying emerging environmental risk factors is crucial for understanding the additional mechanisms that contribute to gastric carcinogenesis. In recent years, environmental pollution has emerged as a potential risk factor for the development of GC, as several epidemiological studies have reported higher incidence and mortality rates of this neoplasm in populations living in highly polluted areas, particularly due to particulate matter groups [2].

Although PM is typically associated with pulmonary pathologies, its ability to enter the body via multiple routes makes gastric tissue a target of ongoing exposure. The combination of mucociliary clearance, which redirects inhaled particles toward the esophagus, and the direct ingestion of particles deposited in contaminated food or water ensures a sustained presence of contaminants in the gastric lumen [59,65]. This route of systemic exposure suggests that the stomach is not only exposed to carcinogens voluntarily ingested but is also vulnerable to air pollutant exposure, thereby integrating air pollution as an exogenous risk factor for this neoplasm. Given this scenario, it is crucial to elucidate the molecular mechanisms by which this pollutant exerts adverse effects on the gastric epithelium and to clarify its role as an emerging risk factor in gastric carcinogenesis.

In GC, the transition from healthy gastric mucosa to adenocarcinoma typically involves several histopathological events, many of which are driven primarily by a persistent inflammatory microenvironment. In this context, the NF-κB, MAPK, and JAK/STAT signaling pathways do not act as isolated events but rather function as critical molecular mediators that translate chronic stress into oncogenic genomic responses. Sustained activation of these pathways orchestrates the transcription of multiple pro-inflammatory mediators and enzymes, including COX-2, whose overexpression is a distinctive marker of gastric tumor progression, as it can promote proliferation, angiogenesis, resistance to apoptosis and even the process of EMT. Therefore, dysregulation of these inflammatory pathways can establish conditions necessary for the survival of damaged cells, driving cell transformation, as well as having implications for disease progression [174,175,176].

Within this framework, the present review synthesizes multiple lines of evidence demonstrating the capacity of PM to trigger and sustain these canonical inflammatory signaling pathways. Highlighting that activation of these pathways by PM leads to a sustained increase in proinflammatory cytokines and, therefore, to the establishment of a persistent inflammatory microenvironment. However, an inherent limitation of the current evidence is that most findings derive from lungs, skin, endothelial tissue, or brain models rather than directly from gastric tissue. This lack of specific studies not only highlights the limited research on the air-stomach axis but also suggests an underestimation of PM true impact on this organ. If it is confirmed that these pollutants induce analogous changes in the stomach, the pattern would be consistent with the mechanisms of traditional risk factors, such as Hp or tobacco, positioning chronic inflammation as the central axis through which environmental and conventional factors converge.

The evaluation of PM as mediators of inflammatory signaling is a priority objective for understanding its impact on the etiology of GC. Inflammation is considered a central component that interacts with virtually all hallmarks, facilitating and enhancing many of them, such as sustained proliferation and evasion of apoptosis, through mediators such as IL-8, IL-6, and TNF-α which catalyze multiple anti-apoptotic and pro-proliferative genes [177,178]. By suppressing programmed cell death mechanisms and blocking caspase activity, a niche is established that favors tumor progression, allowing cells with genomic alterations to survive and facilitating the progression from preneoplastic stages to malignant neoplasms. That is why we propose that chronic inflammation resulting from PM exposure could also promote secondary genomic damage and profound alterations in the gastric epithelium and microenvironment.

Beyond inflammation, PM has been shown to induce oxidative stress, DNA damage, genomic instability, mutations, and epigenetic alterations, all of which may contribute to gastric carcinogenesis [179,180,181,182]. Given the highly heterogeneous genetic landscape of GC, PM-induced cellular stress may affect multiple molecular pathways commonly dysregulated during gastric tumorigenesis. In GC, genes such as *TP53*, *CDH1*, and *PIK3CA* are frequently mutated or epigenetically altered and play critical roles in the regulation of cell cycle progression, DNA repair, apoptosis, epithelial integrity, and maintenance of genomic stability. Therefore, PM-associated molecular damage could potentially contribute to gastric carcinogenesis by promoting alterations in these key oncogenic and tumor suppressor pathways within gastric tissues. Experimental evidence has demonstrated that PM exposure can impair p53 signaling through epigenetically mediated reduction of *TP53* expression or induction of *TP53* mutations, thereby compromising cellular responses to genotoxic stress [181,183]. In addition, PM exposure has been associated with epigenetic silence of *CDH1* and activation of oncogenic pathways, including PI3K/AKT signaling, alterations that may favor epithelial dysfunction, loss of genomic integrity, and the acquisition of malignant phenotypes during gastric carcinogenesis [184,185,186].

Importantly, although most evidence supporting these molecular alterations derives from respiratory exposure models, direct evidence demonstrating these specific effects in human gastric tissue remains limited. Therefore, confirming whether similar PM-induced molecular mechanisms occur within the gastric microenvironment could reveal a previously underrecognized pathway involved in gastric carcinogenesis, characterized by genomic instability, tumor suppressor silencing, and activation of pro-survival signaling pathways. This important knowledge gap highlights the need for studies specifically designed to validate these mechanisms in gastric cancer cohorts. Altogether, the available evidence suggests that PM-mediated carcinogenicity likely results from a complex interplay among chronic inflammation, oxidative stress, somatic mutations, and epigenetic dysregulation affecting critical pathways involved in gastric oncogenesis.

In this context, the composition of PM emerges as a critical factor in explaining its carcinogenic potential. The presence of redox-active transition metals (e.g., Fe, Cu, or Ni) and PAHs is a common characteristic of PM globally, regardless of its geographic origin [35,187]. These components can induce oxidative stress directly through redox reactions or indirectly through the metabolism of PAHs by biotransformation enzymes, thereby amplifying the inflammatory response [188,189]. Therefore, although the absolute potency of PM can vary between sites due to differences in their composition and concentration, the activation of pathways related to oxidative stress and inflammation could represent a conserved mechanism, suggesting that the observed effects are biologically relevant beyond the specific context of exposure. In this regard, it is crucial to consider not only particle size but also the different routes by which PM can exert their biological effects in extrapulmonary organs, including the gastrointestinal tract.

While most epidemiological studies have focused on fine and ultrafine particles, primarily PM_2.5_ and PM_1_ due to their greater systemic penetration capacity and more pronounced biological effects, PM_10_ particles also represent a relevant component that should be considered. Due to their larger size and mucociliary clearance mechanisms, PM_10_ particles can be more easily swallowed after being deposited in the upper respiratory tract, increasing the likelihood of direct contact with the gastric epithelium. This process suggests that PM_10_ could play a significant role in the direct exposure of the stomach to inhaled pollutants and, therefore, in associated pathological processes, including gastric cancer.

The high probability of co-exposure to PM and factors such as H. pylori or smoking in urban populations positions air pollution as a critical determinant of gastric cancer. It is proposed that PM potentiates the baseline inflammatory state, interacting with lesions caused by other agents to promote accelerated malignant progression. However, the biological synergies among these elements have not yet been integrated into risk models. Given that these factors share similar effector pathways, including inflammation, oxidative stress, and DNA damage, their interactions likely amplify oncogenic signaling, reducing the latency period in tumor development.

The inclusion of PM in the etiological map of GC provides a necessary perspective on how environmental factors shape tumor progression. Integrating environmental burden into predictive models will enable the study of the biological interactions between pollutants and classical carcinogens, a priority given the global increase in this disease. The transition to precision epidemiology, which integrates personal monitoring with molecular analysis, is fundamental to identifying the factors that trigger gastric carcinogenesis at the individual level. Thus, clarifying the contributions of these pollutants constitutes a necessary scientific basis for promoting environmental regulations that prioritize long-term protection of at-risk populations.

## 10. Materials and Methods

The present review integrates available epidemiological evidence with experimental data describing key signaling pathways to provide a comprehensive framework for interpreting the impact of PM exposure on gastric malignant transformation. The literature search focused on original epidemiology, in vivo and in vitro studies, available in PubMed/MEDLINE, Scopus, SciELO and Google Scholar databases between January 2010 and March 2026. The search strategy was designed using a combination of controlled terms (MeSH/DeCS) and keywords using Boolean operators (AND, OR). The main descriptors included terms related to the pathology (“Gastric cancer”, “Gastric carcinogenesis”, “Gastric cancer risk”), the exposure agent (“particulate matter”, “PM”, “PM_10_”, “PM_2.5_”, “PM_0.1_”), and the biological mechanisms of interest (“Inflammation and gastric cancer”, “Inflammation and PM”, “NF-κB”, “MAPKs”, and “JAK/STAT”).

## 11. Conclusions

This review integrates emerging environmental exposures into the gastric carcinogenesis framework. Epidemiological evidence consistently links high PM levels to increased GC incidence and mortality. Mechanistically, PM acts as a microenvironment modulator by sustainably activating inflammatory pathways (NF-κB, MAPK, JAK/STAT) also triggered by traditional risk factors, thereby intensifying the pre-existing inflammatory burden and promoting malignant progression. The bioavailability of PM-associated toxicants under gastric conditions confirms the stomach as a direct target organ. This convergence is critical in urban settings, where chronic pollution overlaps with other risk factors. Consequently, PM must be integrated into GC risk models. Addressing this environmental dimension is essential for a comprehensive understanding of the disease and for developing preventive public health policies in the context of global urbanization and deteriorating air quality.

## Data Availability

No new data were created or analyzed in this study. Data sharing is not applicable to this article.

## Abbreviations

The following abbreviations are used in this manuscript:

PM	Particulate Matter
GC	Gastric Cancer
Hp	Helicobacter pylori
